# The development of a novel, multilingual IBD knowledge questionnaire for Asian patients with inflammatory bowel disease

**DOI:** 10.1186/s12876-023-02817-0

**Published:** 2023-05-25

**Authors:** Nik Razima Wan Ibrahim, Mahmoud Danaee, Xin-Hui Khoo, Suresh Sithambaram, Shahreedhan Shahrani, Alex Hwong-Ruey Leow, Jo-Ven Chang, John Francis Mayberry, Ida Normiha Hilmi

**Affiliations:** 1 Division of Gastroenterology and Hepatology, Department of Medicine, Faculty of Medicine, University Malaya, Lembah Pantai, Kuala Lumpur 59100 Malaysia; 2 Department of Social Preventive Medicine, Faculty of Medicine, University Malaya, Kuala Lumpur, Malaysia; 3 Manipal Hospitals, Port Klang, Selangor Malaysia; 4 Nuffield Hospital, Leicester, UK

**Keywords:** Inflammatory bowel disease, Knowledge assessment, Validity, Disease education, Questionnaire design

## Abstract

**Background:**

Inflammatory bowel disease is an uncommon disease in developing nations whereby patient’s knowledge on the disease may be limited. The CCKNOW questionnaire, a widely known questionnaire to assess patient’s knowledge on the disease, may be too complex to comprehend for patients in developing countries. The aim of this study is to develop a new tool known as AIBDKQ questionnaire to evaluate the local inflammatory bowel disease patient’s knowledge.

**Methods:**

This was a prospective study carried out in four phases. In phase 1, three gastroenterologists with expertise in IBD generated a total of 21 questions related to the general knowledge of the disease in the English language. Phase 2 involved content and face validity whereby the questions were further validated by other gastroenterologists. In phase 3, the validated questions were translated into three languages namely Malay, Mandarin and Tamil which are commonly used in Malaysia. In phase 4 (statistical validity), administration of the questionnaires to patients and hospital staff were conducted to assess the construct validity, discriminative ability, predictive validity and reliability of the questionnaires.

**Results:**

A total of 21 questions were generated initially. Further evaluation indicated that 20 items had adequate kappa and content validity index for relevance (CVI: 0.714 to 1, Kapp: 0.645 to 1) and clarity (CVI: 0.714 to 1, Kapp: 0.645 to 1). The questionnaires in four languages were administered to 213 patients to assess the construct validity. Six items were removed (three for low communality, one for small loading factors, two for cross loading), resulting in 16 final questions. Assessment with 34 hospital staff involving nurses, doctors and clerks showed significant differences in knowledge between the groups (F = 14.007, p < 0.001) and were able to discriminate doctors from nurses and clerks. Another group of 18 hospital staff administered with AIBDKQ and CCKNOW questionnaires showed a Pearson’s correlation coefficient of 0.8 indicating strong correlation and concurrent predictive validity between the two questionnaires. Final assessment with 38 patients for reliability assessment revealed high intraclass correlation of the questionnaire among the four languages.

**Conclusions:**

The AIBDKQ has an excellent discriminant ability and internal consistency with a strong correlation when compared to the standard CCKNOW questionnaire.

**Supplementary Information:**

The online version contains supplementary material available at 10.1186/s12876-023-02817-0.

## Introduction

Inflammatory bowel disease (IBD), comprising of ulcerative colitis (UC) and Crohn’s disease (CD), is a chronic idiopathic inflammatory condition with intestinal and extra-intestinal features. IBD is a well-known disease in the Western world with a current prevalence of about 0.5% [1]. IBD has been relatively rare in developing nations. However, over the past few decades, newly industrialized countries in Asia, South America and the Middle East have documented the emergence of IBD. In Malaysia, the mean incidence of IBD has increased steadily in the past decades, with prevalence rates of 23.0, 15.67 and 7.36 per 100,000 persons for IBD, UC and CD respectively [2, 3]. When stratified according to the main multi ethnic groups in Malaysia, the highest prevalence of IBD was in Indians, followed by the Malays and Chinese [2, 3].

IBD is characterized by alternating periods of remission and relapse of active symptomatic disease from the moment of diagnosis for the rest of life. Since the majority of patients are diagnosed at an early age, they need to learn how to cope with problems arising from the disease. However, due to the lower incidence and prevalence rates in Asian countries compared to Western countries, there is a lack of social support and resources to help patients with IBD, in which the patient’s knowledge on the disease may not be sufficient. A greater understanding of their chronic illness may help in long term management. Education on the disease has been shown to have a positive impact on medicine adherence and also influence decision making in terms of important issues such as family planning and career [4, 5]. Higher levels of knowledge have also been shown to reduce health care costs in patients with IBD [6].

In advanced Western nations, some studies have been performed to assess the knowledge of the disease amongst IBD patients. The CCKNOW (Crohn’s and Colitis Knowledge) is a self-administered 24 item questionnaire, created in 1998 by Eaden et al. which provides a valuable index of overall knowledge amongst IBD patients [7]. The CCKNOW is to date, the most widely used questionnaire throughout the world. In the United Kingdom and the United States of America, the mean scores were 12.4 (UC) and 11.5 respectively [8]. In contrast however the scores in developing countries were much lower, i.e. 4.65 in Iran [9] and 6.86 in Sri Lanka [10]. Although this may reflect the overall knowledge of the population, it is also possible that cultural suitability poses a barrier for patients to fully understand and interpret the more complex questionnaires, even if it is translated into the local language. In addition, CCKNOW was developed more than two decades ago and may no longer fully reflect current issues.

Malaysia comprises of a multiracial ethnic community which includes mainly Malays, Chinese and Indians. Some patients undeniably will feel more comfortable in their native languages especially when answering questions about their understanding of the disease. Therefore, there is a need for a simpler and more readable, updated set of questionnaires, which would be easier for local patients to comprehend.

This paper describes the development and validation of a new instrument, the Asian IBD Knowledge Questionnaire (AIBDKQ). The questionnaire was subsequently translated into three languages commonly used in Malaysia and which can be used in IBD patients to assess their knowledge on the disease.

## Methodology

The AIBDKQ was developed in four main phases as described below:

- item generation.
- assessment of logical validity.
- translation of the questionnaire.
- and finally assessing its statistical validity.

The protocol of the study was reviewed and approved by the Medical Research Ethics Committee of UMMC (MREC ID NO: 201964-7490). Informed consent was obtained from all participants (individuals above 18) prior to administering the questionnaires.

### Phase 1 – item generation

The idea of generating questions came from IH. She then selected two more IBD experts (JM and AL) to come up with appropriate questions for the formation of AIBDKQ. The items were generated through related literature and research as well as expert’s observations to test the patient’s general knowledge on IBD. The questions were created and discussed with experts individually to assess its suitability for the questionnaire. A total of 21 questions were generated by three IBD experts in the English language.

### Phase 2 – logical validity: content and face validity

The 21 questions which were generated in phase 1 were given to a panel of experts consisting of seven gastroenterologists to assess their relevancy (relevant to the concept or construct being measured) and clarity (wording of the items) as well as any specific comments or feedback on the items.

Each of the items were rated based on a 4-point scale, with the least point being not relevant and the maximum point being highly relevant. All items were subsequently calculated for the kappa value (Kapp) and its content validity index (CVI). The cut-off point taken for an accepted Kapp and CVI was 0.7. Any items with a score less than 0.7 was removed from the final questionnaire.

### Phase 3 – translation of the questionnaire

The questions were then further translated to three different languages that are commonly used in Malaysia, namely Malay, Mandarin and Tamil. The process of translation was done according to WHO guideline “Process of translation and adaptation of instruments” [11].

Forward translation into Malay was done by NW, gastroenterologist and back translation to English was done by ShS, gastroenterologist. Translation into Mandarin was done by JC, a medical officer and back translation to English was done AL, consultant gastroenterologist. Translation into Tamil was done by TG, a professional Indian translator and back translation was done by SS, consultant gastroenterologist. All translation work was done by people who were native and proficient in both languages. All the translated items were then compared with original English version. The process took place with multiple discussions with the original developers to ensure preservation of the meaning of each item. The translated items were further tested on nine subjects who were native to the language (three subjects for each language) and who could give feedback on the choice of words used for its understandability, interpretation and cultural relevance of the translation. Any discrepancies were amended. The outcome of this was subjected to a final review to highlight and correct any typographic or grammatical errors. A final report was done at the end of the process documenting the development of each translation as detailed in Table 1.

**Table 1:** Translation Process of AIBDQ Questionnaire

Steps	Process	Key person involved
Step 1	Preparation	Personnel were identified for the translation process.
	Forward translation to the target language (Malay, Mandarin, Tamil)	Done by personnel with the languages that were native to them
Step 2	Back translation done to English language	Done by gastroenterologists who were fluent in both languages
Step 3	Back translation review and harmonisation	Done by everyone in the expert committee (authors of the paper)
Step 4	Cognitive debriefing	Nine healthy subjects (three for each translation)
Step 5	Review of cognitive debriefing and proof reading	Expert committee (authors of the paper)
Step 6	Final report	NW

### Phase 4 – statistical validity (psychometric assessment)

#### Sample population and sampling

The estimated sample population was 400 based on the number of patients attending outpatient IBD clinic in University Malaya Medical Centre (UMMC). The hypothesized frequency of the outcome factor is unknown, hence it was set at 50%. Using the OpenEpi open-source calculator, the sample size needed to give 95% confidence interval was calculated as 197 [12].

#### Construct validity

Patients attending IBD clinic in UMMC were then asked to fill up the questionnaire. The patients were approached and interviewed by single interviewer (NW). Subjects included were all IBD patients attending the IBD clinic. They were briefed prior to administering the questionnaire and subsequently assessed for their understanding. The patients were asked to select a version of the questionnaire with the language that they were most proficient and comfortable to use. All the questionnaires were completed before their clinic consultation.

#### Discriminative ability

Discriminative ability of the questionnaire was validated using the English version of the questionnaire in three occupational groups with different levels of IBD-related knowledge. Subjects chosen to answer the questionnaire comprised of nurses, junior doctors and clerks selected from UMMC. All subjects chosen were adequately proficient in basic written and spoken English language. All subjects were instructed to fill up the questionnaire to the best of their knowledge and the scores derived from the three groups were collected and compared.

#### Predictive validity

To measure the questionnaire’s predictive validity, a correlation between the questionnaire were compared against the CCKNOW and AIBDKQ. Another group of subjects from three occupational groups, namely nurses, junior doctors and clerks, were asked to fill up both the English version of the developed questionnaire and the CCKNOW. Both questionnaires were given together at the same setting.

#### Reliability of the instrument

Reliability of the questionnaire were tested on a small group of patients with questionnaires in all of the four languages. Subjects included were all IBD patients attending the IBD clinic. The patients were approached and also interviewed by a single interviewer (NW). They were briefed prior to administering the questionnaire and subsequently assessed for their understanding with regards to the aim of the study, consent, and how to fill in the questionnaires. The patients were asked to select a version of the questionnaire in the language that they were most proficient and comfortable to use. All the subjects were asked to fill up the questionnaire twice; at the first meeting and the second one done within two weeks apart. The second questionnaire was sent to patients via post or electronically via email. The subjects were also reminded not to do any reading or take further courses regarding IBD during the interim. The reliability was done based on the test-retest results collected after two weeks.

### Data analysis

The data analysis was done using the SPSS version 25.0 (SPSS Inc., Chicago, IL, USA). The numerical data are presented as mean and standard deviation. This phase included four methods for psychometric assessment including construct validity, discriminative validity, predictive validity and reliability of the instrument. Construct validity of the questionnaire was analysed using exploratory factor analysis (EFA). EFA is a data analysis technique that determines the structure of factors to be explored [13]. It was used to figure out the number of dimensions between relationship of items and factors. Discriminative validity was done using one-way ANOVA. Predictive validity was evaluated using Pearson or Spearman rank-correlation coefficient. Internal consistency of the questionnaire was evaluated using Kuder Richardson Reliability Coefficient (KR20) with STATTODO (online statistical package). A value of P < 0.05 is considered statistically significant.

## Results

### Phase 1 – item generation

The three IBD experts came up with a total of 30 questions. Items included were questions regarding general knowledge of disease, its symptoms, treatment and disease complication. Selection of the questions were done together with all the authors. There were several meetings, mostly online and each of the questions created were discussed. The meetings were synchronised by NW.

Suitable questions were selected and the experts finally decided on 21 items. The items consisted of multiple-choice answers and true or false options. Each correctly answered question was given a score of 1. Three questions had sub questions (Question 8, 16 and 17) giving the total score of 32 if all answered correctly, as shown in Table 2.

**Table 2:** Items (Questions) generated

No.	Type	Items/Questions
Q1	Multiple-choice answers	What best describes the disease/condition called inflammatory bowel disease or IBD?
Q2	Multiple-choice answers	What causes IBD?
Q3	Multiple-choice answers	If you have IBD, your children are also at high risk (more than 20%) of developing IBD.
Q4	Multiple-choice answers	The terminal ileum is the last part of the small bowel (where the small bowel and the large bowel are connected)
Q5	Multiple-choice answers	Crohn’s disease occurs only in the large bowel.
Q6	Multiple-choice answers	Ulcerative colitis occurs mainly in the large bowel.
Q7	Multiple-choice answers	The stomach is often affected in IBD.
Q8	True/false options	What are the COMMON symptoms of IBD?
Q8a		Blood in stool
Q8b		Diarrhoea
Q8c		Constipation
Q8d		Abdominal (‘stomach’) pain
Q8e		Headache
Q9	Multiple-choice answers	Patients with IBD can have others organ involvement/inflammation as well such as the eyes, skin and joints.
Q10	Multiple-choice answers	Dairy-based foods (e.g. milk and cheese) need to be completely avoided as these may trigger IBD episodes.
Q11	Multiple-choice answers	Exercise may trigger IBD episodes.
Q12	Multiple-choice answers	IBD can be spread to close contacts so it is important not to share food.
Q13	Multiple-choice answers	Smoking will worsen the symptoms for
Q14	Multiple-choice answers	How do most IBD drugs work?
Q15	Multiple-choice answers	IBD can be cured once you have taken your medication for a long time.
Q16	True/false options	Below are the common side effects of prednisolone:
Q16a		Bone thinning/osteoporosis
Q16b		Chest pain
Q16c		Eye cataract
Q16d		Gum swelling
Q16e		High blood sugar
Q17	True/false options	Known side effects of azathioprine include:
Q17a		Damage to the baby in pregnancy
Q17b		Difficulty in breathing
Q17c		Liver swelling (hepatitis)
Q17d		Low white blood cell count
Q18	Multiple-choice answers	Biologic therapy is a potent drug that is used only for severe disease.
Q19	Multiple-choice answers	What is your understanding on the effect of IBD drugs in pregnancy?
Q20	Multiple-choice answers	Persons with IBD have an increased risk of having colon cancer.
Q21	Multiple-choice answers	Patients with Crohn’s disease can have narrowing of the bowel and rupture of the bowel.
Total items: 32 Total questions: 21		

### Phase 2 – logical validity: content and face validity

Results of expert evaluation by seven gastroenterologists were applied to calculate the CVI and Kapp value to assess the content and face validity. Results indicated that all items had adequate kappa and CVI for the assessment of relevance (CVI: 0.714 to 1, Kapp: 0.645 to 1) and clarity (CVI: 0.714 to 1, Kapp: 0.645 to 1) except for question 4 which had a low CVI and Kapp value for relevance although it has acceptable CVI for clarity, hence was removed from the final questionnaire (Table 3).

**Table 3:** Relevance and clarity of the questions (content and face validity)

Questions	Relevance		Clarity	
	CVI	Kappa	CVI	Kappa
Q1	1.000	1.000	0.857	0.849
Q2	0.857	0.849	1.000	1.000
Q3	1.000	1.000	1.000	1.000
**Q4**	**0.429**	**0.214**	**0.714**	**0.658**
Q5	1.000	1.000	0.857	0.849
Q6	1.000	1.000	0.714	0.658
Q7	0.857	0.849	1.000	1.000
Q10	1.000	1.000	0.857	0.849
Q13	0.857	0.849	0.857	0.849
Q14	1.000	1.000	0.857	0.849
Q15	1.000	1.000	0.857	0.849
Q18	1.000	1.000	1.000	1.000
Q19	1.000	1.000	0.857	0.849
Q21	1.000	1.000	0.857	0.849
Q8	1.000	1.000	0.857	0.849
Q11	1.000	1.000	1.000	1.000
Q12	0.857	0.849	0.714	0.658
Q20	1.000	1.000	1.000	1.000
Q9	1.000	1.000	0.857	0.849
Q16	1.000	1.000	0.857	0.849
Q17	1.000	1.000	1.000	1.000
Q8	0.714	0.658	1.000	1.000
Q16	0.857	0.849	1.000	1.000
Q17	0.857	0.849	0.857	0.849

### Phase 3 – translation of the questionnaire

Following the extensive translation process of the initial English version, three translated versions were produced in Malay, Mandarin and Tamil languages. Final version of the questionnaires in the three languages were supplemented in Supplementary Content 1.

### Phase 4 – statistical validity and reliability (psychometric assessment

#### Phase 4 − 1 - construct validity

All the 4 versions of the questionnaire were then administered to patients attending the IBD clinic in UMMC.

A total of 213 patients filled up the questionnaire during their visit at the clinic. From the data collected, exploratory component analysis was done on knowledge items to see if all of the variables in this study had sufficient construct validity. The factor structure of 31 items related to knowledge was determined using EFA. Initial analysis indicated that three items (Q3, Q10 and Q19) had a low communality were below the threshold of 0.3, therefor these items were removed from the analysis.

**Fig. 1:**
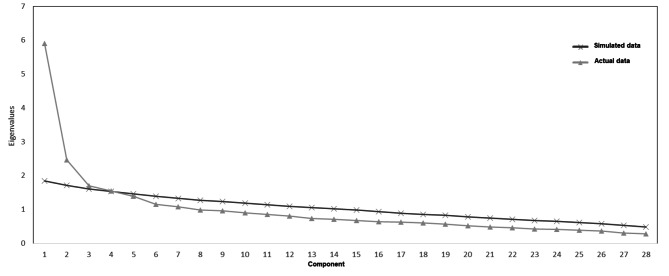
Screen Plot and Parallel Analysis for Items Related to Knowledge

Prior to conducting the EFA for the evaluation of construct validity of the research questionnaire, it was necessary to determine the number of components (factors) for knowledge. Parallel analysis is a Monte Carlo simulation method that helps scholars in defining the number of factors to retain in EFA. In this study, parallel analysis was done based on this method using an online software STATTODO (www.stattodo.com). The parallel analysis for 28 items of knowledge indicated that the eigenvalue for the fourth extracted factor was nearly equal to the eigenvalue that could be expected by chance (λ = 1.536) (Fig. 1). The results of this parallel analysis indicated that only four factors have eigenvalues greater than what can be expected by chance and suggested that four factors could be extracted from the data and therefore in the next step for EFA the number of extracted components was considered as five factors.

The factorability of correlational items was assessed using a number of well-known criteria. To begin, the Kaiser-Meyer-Olkin measure of sample adequacy was 0.814, which was significantly higher than the required value of 0.6, and Bartlett’s test of sphericity was significant (χ^2^_(378)_ = 1531.79, p < 0.001) and all communalities in the current investigation were above the threshold, and all loading factors were greater than 0.4 except for one items Q8d, which was removed from the final questionnaire. EFA was performed based on the results of parallel analysis, and the number of components was determined to be four based on these findings.

The results after Varimax rotation showed that the first factor which is related to “General knowledge of disease and pathogenesis” which explained 11.83% of the variance including seven items. The second factor with six items was related to “Symptoms and triggers of IBD” had 11.19% of the variance. Result of factor analysis indicated that the third component with seven items was related to “Likely side effects of treatment and long-term disease complication” which explained 9.502% of the variance. The fourth components which explained 8.99% of the variance with five items was about measuring “Unlikely side effects of treatment” with five items. Total variance explained by these four components was 41%. Three items (Q1, Q8d and Q8e) were cross loaded and therefore were removed from the final questionnaire (Table 4).

**Table 4:** Factor loadings based principal component analysis with Varimax Rotation for 28 items related to knowledge (n = 213)

Item	Component			
	1	2	3	4
Q5	0.732			
Q14	0.655			
Q13	0.633			
Q2	0.532			
Q18	0.528			
Q21	0.496			
Q15	0.494			
**Q1**	**0.470**	**0.43**		
**Q8d**	**0.375**	**0.356**		
Q12		0.698		
Q11		0.649		
Q8b		0.639		
Q8a		0.606		
Q20		0.454		
Q7		0.400		
Q16e			0.656	
Q9			0.562	
Q16c			0.559	
Q17d			0.541	
Q17c			0.537	
Q16a			0.506	
Q6			0.412	
Q16b				0.725
Q17b				0.717
Q16d				0.667
Q17a				0.632
Q8c				0.509
**Q8e**		**0.395**		**0.495**
Eigenvalues	3.314	3.134	2.661	2.519
% of Variance	11.835	11.194	9.502	8.998

Therefore, a total of seven items were removed from the original 32 items at this stage. One item was based on logical validity (Q4) and six from construct validity (Q3, Q10, Q19, Q8d, Q1 and Q8e). The final version of the questionnaire now had 16 questions. Three questions had sub questions, which consisted of a total of 12 sub questions (8a, 8b, 8c, 16a, 16b, 16c, 16d, 16e, 17a, 17b, 17c, 17d), giving the total score of 25 (Table 5). The final version of the questionnaire was named as the Asian Inflammatory Bowel Disease Knowledge Questionnaire (AIBDKQ) (Supplementary Content 1).

**Table 5:** Final outcome and scoring system of Asian Inflammatory Bowel Disease Knowledge Questionnaire (AIBDKQ)

Question	Outcome	Score
Q1	Removed in phase 4b	
Q2	Retained	1
Q3	Removed in phase 4b -low communality	
Q4	Removed in phase 2 - low relevance for content	
Q5	Retained	1
Q6	Retained	1
Q7	Retained	1
Q8 Q8a Q8b Q8c Q8d Q8e	Retained Retained Retained Removed in phase 4b Removed in phase 4b	1 1 1
Q9	Retained	1
Q10	Removed in phase 4b -low communality	
Q11	Retained	1
Q12	Retained	1
Q13	Retained	1
Q14	Retained	1
Q15	Retained	1
Q16		
Q16a	Retained	1
Q16b	Retained	1
Q16c	Retained	1
Q16d	Retained	1
Q16e	Retained	1
Q17		
Q17a	Retained	1
Q17b	Retained	1
Q17c	Retained	1
Q17d	Retained	1
Q18	Retained	1
Q19	Removed in phase 4b -low communality	
Q20	Retained	1
Q21	Retained	1
Total items remaining: 25 Total questions: 16		Total score: 25

#### Phase 4 − 2 - discriminative ability

**Fig. 2:**
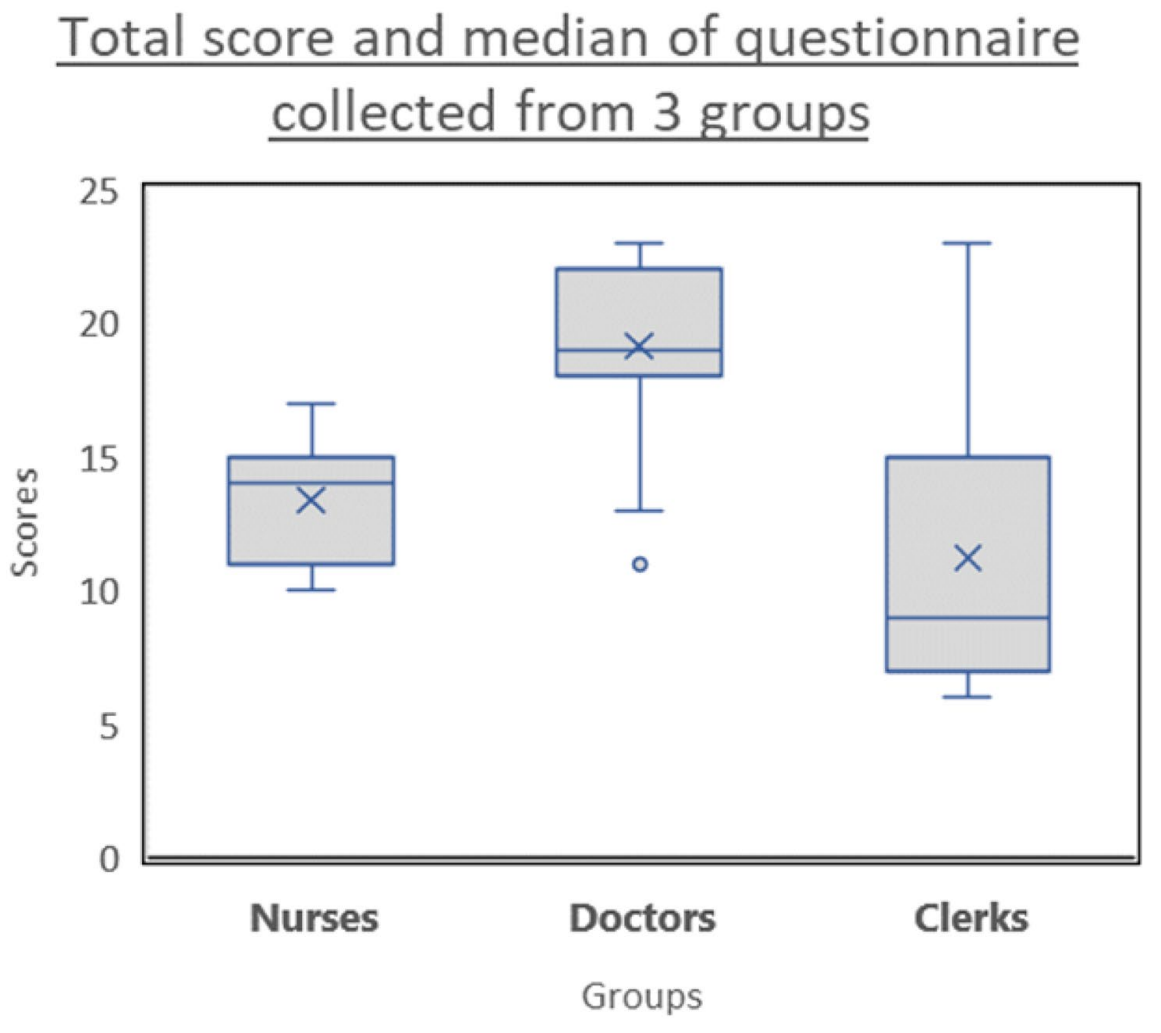
Total score and median of AIBDKQ answered by three groups of subjects with different level of IBD-related knowledge

A total of 34 subjects (11 nurses, 15 junior doctors and 8 clerks) from the UMMC answered the English version AIBDKQ questionnaire. The mean score for nurses was 13.36, doctors was 19.13 and clerks was 11.22 (Fig. 2). In order to evaluate discriminative validity of the questionnaire, the differences among the three groups of subjects were studied using one-way ANOVA. The result of one-way ANOVA showed that there were significant differences among groups for the total score of the questionnaire (F = 14.007, p < 0.001). Bonferroni test also was used in order to compare between groups and results showed that there was a significant difference between the doctors and the nurses as well as the clerks whilst there was no significant difference seen between the clerks and nurses (Table 6). This indicates that this new questionnaire was able to show significant differences in knowledge and is able to discriminate between two groups (doctors compared to nurses and clerks) but not between all the groups. This could be partly confounded by the fact that the sample size used was small.

**Table 6:** Pairwise comparison among the three groups of subjects using Bonferroni test

(I) Group	(J) Group	Mean Difference (I-J)	Std. Error	Sig.	95% Confidence Interval	
					Lower Bound	Upper Bound
Nurses	Doctors	-5.77	1.52	0.002	-9.61	-1.92
Nurses	Clerks	2.14	1.72	0.668	-2.21	6.49
Doctors	Clerks	7.91	1.61	0	3.83	11.99

#### Phase 4 − 3 - predictive validity

**Fig. 3:**
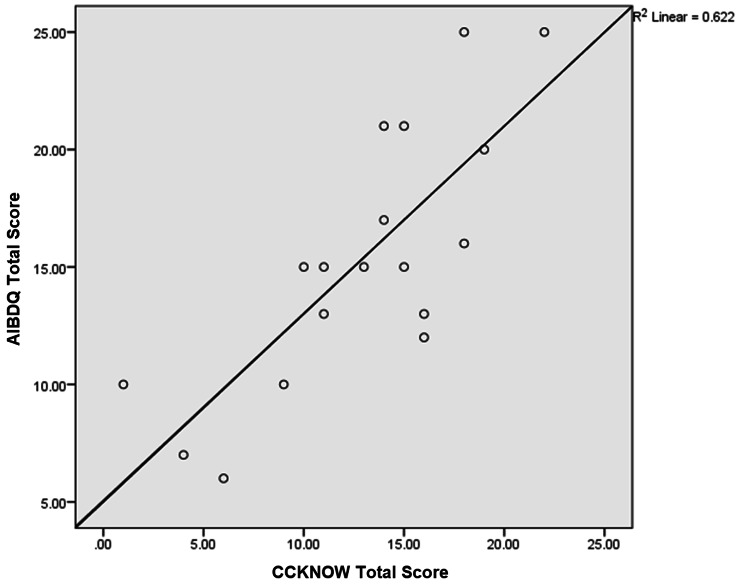
Correlation between AIBDKQ and CCKNOW Questionnaires

To measure the questionnaire’s predictive validity, another group of subjects from three occupational groups, namely nurses, junior doctors and clerks were asked to fill up both the English version of developed AIBDKQ questionnaire and CCKNOW questionnaire. A total of 18 subjects (five nurses, five junior doctors and eight clerks) answered both the questionnaires. The calculated Pearson correlation coefficient between the AIBDKQ and CCKNOW was 0.8. This indicated a very strong correlation and concurrent predictive validity between AIBDKQ and the CCKNOW (Fig. 3).

#### Phase 4–4 - reliability of the instrument

A total of 38 patients from the IBD clinic filled the AIBDKQ questionnaire. The first time was during the clinic visit and subsequently a second time using the same questionnaire after two weeks. The second questionnaire were given either via post or electronically done via email. Ten patients filled up using the English version, ten patients in the Malay version, eight patients in the Mandarin version and ten patients in the Tamil version. Both English and Malay versions were filled by all three races. The Chinese version was filled by Chinese patients only and the Tamil version was filled by Indian patients only.

The overall total scores of each language group were calculated and checked for test-retest reliability. The range of intraclass correlation was 0.967 for English Language, 0.919 for Malay Language, 0.949 for Chinese language and 0.958 for Tamil language (Table 7). The levels indicate high intraclass correlation and all are statistically significant.

**Table 7:** Intraclass correlation coefficient of AIBDQ in different languages

Language	Intraclass Correlation	95% Confidence Interval	
		Lower Bound	Upper Bound
English	0.967	0.865	0.992
Malay	0.919	0.674	0.98
Chinese	0.949	0.744	0.99
Tamil	0.958	0.829	0.989

## Discussion

Little is known about the knowledge of IBD amongst our patients. The disease itself, being rather uncommon in Malaysia makes it difficult for patients to discuss about it amongst themselves. It is widely known that part of improving patient care is educating patients of their chronic illness. Part of the effort to develop an education program is to first understand the gaps in knowledge in the local setting.

This study is the first in the country where we developed a questionnaire as a method to determine the health-related knowledge of IBD patients in Malaysia. Although CCKNOW has been used as a reliable indicator of knowledge, the translated versions of CCKNOW applied to non-native English-speaking patients may still not overcome the barriers in the basic understanding of non-Western cultures [9, 10]. This is well recognized in other conditions as well, such as irritable bowel syndrome (IBS) [14]. It is clear that translated versions of other questionnaires may not always be applicable to be used in all cultures as certain items such as names of body parts or descriptions of complication would be described differently in other populations. Furthermore, it is important to incorporate newer questionnaires to include patient’s knowledge on updated issues of a disease.

Other than our study, several other newer questionnaires in IBD have been developed to address these issues. The ‘IBDKNOW’ was developed in the Korean language by Yoon et al., and was further translated into English [15]. However, there were certain aspects of the language used in questionnaire we felt could be too difficult for our population. Furthermore, there were certain areas, such as patients’ knowledge of drug side effects were not tested in the questionnaire. The study clearly showed a discrepancy of knowledge when comparing the Korean and American populations [16]. Another questionnaire was developed by Danion et al. which incorporated CCKNOW, CCKPNOW (Crohn’s and Colitis Pregnancy Knowledge Score) [17] and another set of new complementary updated questions into a new 64 item questionnaire called the IBD-INFO in the French Language [18]. It has been used by French IBD patients to identify their knowledge gap of their disease.

The aim of this study was to address the need to develop a novel questionnaire for the local community which is culturally adaptable to wordings that are commonly used and understood by local patients. The health literacy in many parts of Asia is also very low [19–23]. We also felt that the CCKNOW and was probably too advanced for the basic level of knowledge in our population and that the questionnaire we developed was comprehensive but basic enough to analyse gaps in knowledge. The questionnaire can provide a baseline for the development of knowledge attitude perception (KAP) questionnaires; both prior and following the implementation of patient educational programmes.

The questionnaires were designed by a panel of experts in IBD as well as experts in questionnaire development to ensure the quality of its content. Several other experts were also consulted to acquire its logical validity. Translation into three other languages were done as these are the 3 main native languages used in our multiracial country. All the steps of translation and back translation were meticulously carried out. Following constructive validity, the final version Asian IBD Knowledge Questionnaire has 16 questions with 12 sub questions giving the total score of 25.

The questionnaires were self-administered, ensuring that patients were given with the same instructions and under the same conditions. All enrolled patients were literate and were able to fill all the questionnaires themselves to prevent bias.

When applied to the three different groups; doctors, nurses and clerical staff, the AIBDKQ showed good discriminant ability with excellent internal consistency as shown by the Kuder Richardson score. Moreover, it has a strong correlation with the standard CCKNOW.

The high interclass correlation seen between all the translated versions confirms that it is a reliable tool and can be applied in other populations where Mandarin and Tamil are spoken although we note that some of the Indians and Chinese in fact preferred to answer the questionnaire in the Malay language since it’s the most widely used language in Malaysia. Another strength of the questionnaire is that the English version of AIBDKQ may be more easily translated to other languages as compared to CCKNOW.

The main limitation of this study is that it is a single centre study with a small number of participants. As with the CCKNOW, the AIBDKQ may not be applicable across all the cultures and populations even within Asia. It would of course, exclude large population such as Korea, Japan and certain parts of India where Tamil is not being used. In addition, the drugs that were asked in the questionnaire may also not be used in other parts of Asia, hence limiting the patient’s ability to complete the questionnaire. However, we feel that the main cultural barriers to understand IBD is probably fairly universal across Asia.

## Conclusion

In view of some of the perceived limitations of available knowledge questionnaires, we have developed a novel questionnaire AIBDKQ which was subsequently translated into three common Asian languages; Malay, Mandarin and Tamil. The AIBDKQ has the discriminant ability to show different levels of IBD-related knowledge with excellent internal consistency and has a strong correlation when compared to the standard CCKNOW questionnaire.

## Electronic supplementary material

Below is the link to the electronic supplementary material.

Supplementary Material 1

## Data Availability

All data generated or analysed during this study are included in this published article and its supplementary information files.

## Abbreviations

IBD: Inflammatory bowel disease
UC: Ulcerative colitis
CD: Crohn’s disease
CCKNOW: Crohn’s and Colitis Knowledge
AIBDKQ: Asian IBD Knowledge Questionnaire
CVI: Content validity index
WHO: World Health Organization
EFA: Exploratory factor analysis
IBS: Irritable bowel syndrome
KAP: Knowledge attitude perception
