# Supplementary material for: The development of a novel, multilingual IBD knowledge questionnaire for Asian patients with inflammatory bowel disease
Source: BMC Gastroenterol. 2023 May 25;23:185. doi: 10.1186/s12876-023-02817-0 (PMC10210279; doi:10.1186/s12876-023-02817-0)
Supplement: Supplementary file 1 — Supplementary Material 1 [file 12876_2023_2817_MOESM1_ESM.docx]

Supplementary Content 1

(A) AIBDQ English version

Asian IBD Knowledge Questionnaire (AIBDQ)

1. What causes IBD? (tick one box)

- It is due to a chronic long-term infection of the bowel
- It is due to a food allergy
- It is due to the body’s own immune system (such white blood cells) attacking the body

1. Crohn’s disease occurs only in the large bowel. (Tick one box)

- True
- False
- Unsure/Don’t know

1. Ulcerative colitis occurs mainly in the large bowel. (Tick one box)

- True
- False
- Unsure/Don’t know

1. The stomach is often affected in IBD. (Tick one box)

- True
- False
- Unsure/Don’t know

1. Below are the COMMON symptoms of IBD? (True or False)

Blood in stool True False

Diarrhoea True False

Constipation True False

1. Patients with IBD can have others organ involvements/inflammation as well such as the eyes, skin and joints. (Tick one box)

- True
- False
- Unsure/Don’t know

1. Exercise may trigger IBD episodes. (Tick one box)

- True
- False
- Unsure/don’t know

1. IBD can be spread to close contacts so it is important not to share food. (Tick one box)

- True
- False
- Unsure/don’t know

1. Smoking will worsen the symptoms for (Tick one box)

- Ulcerative colitis
- Crohn’s disease
- Unsure/Don’t know

1. How do most IBD drugs work? (Tick one box)

- They suppress your immune system
- The remove the bacteria from your gastrointestinal system to prevent inflammation
- They improve your immune system so that it can fight the disease
- Unsure/don’t know

1. IBD can be cured once you have taken your medication for a long time. (Tick one box)

- True
- False
- Unsure/don’t know

1. Below are the common side effects of prednisolone (True or False)

Bone thinning / osteoporosis True False

Chest pain True False

Eye cataract True False

Gum swelling True False

High blood sugar True False

1. Known side effects of azathioprine include (True or False)

Damage to the baby in pregnancy True False

Difficulty in breathing True False

Liver swelling (hepatitis) True False

Low white blood cell count True False

1. Biologic therapy is a potent drug that is used only for severe disease. (Tick one box)

- True
- False
- Unsure/don’t know

1. Persons with IBD have an increased risk of having colon cancer. (Tick one box)

- True
- False
- Unsure/don’t know

1. Patients with Crohn’s disease can have narrowing of the bowel and rupture of the bowel.

(Tick one box)

- True
- False
- Unsure/ Don’t know
